# Tincture of Iodine in Vomiting

**Published:** 1872-10

**Authors:** 


Tincture of Iodine in Vomiting.—Schneider, of Offenburg (*The Doctor*, April, 1872), administered tincture of iodine in doses of ten drops on sugar thrice daily to a patient who was troubled with salivation and vomiting after intermittent fever. Although the vomiting had proved rebellious to all other treatment, it yielded to tincture of iodine.

